# Study protocol for a randomized controlled trial of the effectiveness of adding motivational interviewing or stratified vocational advice intervention to usual case management on return to work for people with musculoskeletal disorders. The MI-NAV study

**DOI:** 10.1186/s12891-020-03475-z

**Published:** 2020-07-28

**Authors:** Britt Elin Øiestad, Fiona Aanesen, Ida Løchting, Kjersti Storheim, Alexander Tingulstad, Tarjei L. Rysstad, Milada C. Småstuen, Anne Therese Tveter, Gail Sowden, Gwenllian Wynne-Jones, Egil A. Fors, Maurits van Tulder, Rigmor C. Berg, Nadine E. Foster, Margreth Grotle

**Affiliations:** 1 Department of Physiotherapy, OsloMet – Oslo Metropolitan University, St. Olavs plass, 0130 Oslo, Norway; 2 Research and Communication Unit for MSK Health (FORMI), Oslo University Hospital and Oslo Metropolitan University, Oslo, Norway; 3 Department of Physiotherapy, Research and Communication Unit for MSK Health (FORMI), Oslo University Hospital, Oslo Metropolitan University, Oslo, Norway; 4 Department of Nursing and Health Promotion, Oslo Metropolitan University, Oslo, Norway; 5 National Advisor Unit on Rehabilitation in Rheumatology, Department of Rheumatology, Diakonhjemmet Hospital, Oslo, Norway; 6 School of Primary, Community & Social Care, Keele University, Keele, UK; 7 Connect Health, Newcastle upon Tyne, UK; 8 Faculty of Medicine and Health Sciences, School of Primary, Community and Social Care, and School of Nursing and Midwifery, Keele University, Keele, UK; 9 General Practice Research Unit, Department of Public Health and Nursing Faculty of Medicine and Health Sciences, Norwegian University of Science and Technology (NTNU), Trondheim, Norway; 10 Department Health Sciences, Faculty of Science, Vrije Universiteit Amsterdam, Amsterdam, the Netherlands; 11 Norwegian Institute of Public Health, Oslo, Norway; 12 Department of Community Medicine, The University of Tromsø, Tromsø, Norway

**Keywords:** MSK disorders, Vocational interventions, Motivational interviewing, Sick leave, Return to work, Health economics, Randomized controlled trial

## Abstract

**Background:**

Little research exists on the effectiveness of motivational interviewing (MI) on return to work (RTW) in workers on long term sick leave. The objectives of this study protocol is to describe a randomized controlled trial (RCT) with the objectives to compare the effectiveness and cost-effectiveness of usual case management alone with usual case management plus MI or usual case management plus stratified vocational advice intervention (SVAI), on RTW among people on sick leave due to musculoskeletal (MSK) disorders.

**Methods:**

A multi-arm RCT with economic evaluation will be conducted in Norway with recruitment of 450 participants aged 18–67 years on 50–100% sick leave for > 7 weeks due to MSK disorders. Participants will be randomized to either usual case management by the Norwegian Labour and Welfare Administration (NAV) alone, usual case management by NAV plus MI, or usual case management by NAV plus SVAI. Trained caseworkers in NAV will give two MI sessions, and physiotherapists will give 1–4 SVAI sessions depending upon risk of long-term sick leave. The primary outcome is the number of sick leave days from randomization to 6 months follow-up. Secondary outcomes are number of sick leave days at 12 months follow-up, time until sustainable RTW (≥4 weeks of at least 50% of their usual working hours) at 12 months, proportions of participants receiving sick leave benefits during 12 months of follow-up, and MSK symptoms influencing health at 12 months. Cost-utility evaluated by the EuroQoL 5D-5L and cost-benefit analyses will be performed. Fidelity of the interventions will be assessed through audio-recordings of approximately 10% of the intervention sessions.

**Discussion:**

The results from this RCT will inform stakeholders involved in supporting RTW due to MSK disorders such as staff within NAV and primary health care.

**Trial registration:**

ClinicalTrials.gov ID: NCT03871712 registered March 12th 2020.

## Background

In Norway, musculoskeletal (MSK) disorders present the greatest burden to future health and welfare services [1, 2]. The high economic costs related to sick leave include productivity loss and use of health care services. Importantly, through the Norwegian Labour and Welfare Administration (NAV), the welfare system in Norway covers 100% of the worker’s salary up to 52 weeks of sick leave after the first 16 days, which are paid by the employer.

To address the substantial costs related to sick leave, effective interventions targeting obstacles to return to work (RTW) are needed. In a best evidence synthesis of 94 systematic reviews with half of the studies exploring MSK disorders [3], risk factors associated with poorer RTW outcomes were older age, being female, higher pain or disability, depression, higher physical work demands, previous sick leave, unemployment, and activity limitations. Better RTW outcomes were found in people with higher education and socioeconomic status, higher self-efficacy beliefs, optimistic expectations for recovery, lower severity of the injury/illness, better RTW coordination and in those receiving multidisciplinary interventions involving the workplace and key stakeholders.

There is strong evidence that interventions with a health focus, service coordination involving the workplace, and work modifications reduce the proportion of people on sick leave [4]. Motivational interviewing (MI) [5] is an intervention that targets behavior change that has been suggested to be useful in an RTW context [6]. A systematic review including five randomized controlled trials (RCTs) reported weak evidence for the effectiveness of MI to facilitate RTW, particularly for people with less serious conditions and short work absences [7]. A recent systematic mapping review by our research group [8] with the objective of mapping all types of empirical research on MI as a method to help people with MSK disorders return to work revealed only three papers from two RCTs: A Norwegian RCT with high risk of bias showed now effect of a brief intervention including MI on RTW for 89 disability pensioners with back pain [9], and a Canadian cluster RCT with low risk of bias including 728 claimants with chronic musculoskeletal disorders, showed that providing MI in addition to usual rehabilitation increased RTW both at discharge and at 1-year follow-up [10, 11].

Providing vocational interventions to all people having any period of sick leave, that comprises individualized cognitive, affective and behavioral approaches, would require enormous resources. Using a stratified approach in which only individuals at high risk for long-term sick leave are targeted with the intervention, could be more efficient. *Stratified care* is one approach with which to improve outcomes among people with MSK disorders. By way of example, the STarT Back trial was the first RCT to show that stratified care based on matching treatment to low back pain patients’ risk of persistent disabling pain (low, medium or high risk) resulted in better patient outcomes including fewer days lost from work, at less cost for the UK healthcare system and UK society [12, 13]. Furthermore, a recent RCT [14] compared the difference in the number of sickness absence days between a targeted intervention that emphasized communication (MI and problem-solving skills for the patient and their work supervisor) versus treatment as usual in people with back pain at high risk of persistent pain. The targeted intervention resulted in fewer days off work, fewer health care visits and better perceived health. Despite promising results for low back pain, to date there are few studies on stratified care for the broader group of people with MSK disorders. Several tools have been developed with which to assess MSK pain patients’ risk of persistent disabling pain, for instance the Keele STarT MSK tool [15], and long-term work loss [16]. A *stepped care* approach to facilitate RTW was investigated in the Study of Work and Pain (SWAP) cluster RCT in the UK [17]. General practices were randomized to either offer usual care or the SWAP intervention in addition to usual care. Patients consulting general practices in the intervention arm with MSK pain who were absent from work or struggling at work were offered access to a vocational advice service, whereby vocational advisors provided a case-managed stepwise intervention, starting with brief telephone assessment and advice, addressing obstacles to RTW [18]. The SWAP trial showed that participants in the vocational advice arm had fewer days off work compared with those having usual care alone.

There is lack of research of the effectiveness of MI and vocational advice interventions in people on long term sick leave due to MSK disorders. The objectives of this RCT are thus to compare the effectiveness and cost-effectiveness of usual case management by NAV alone with usual case management by NAV plus MI or usual case management by NAAV plus stratified vocational advice intervention (SVAI), on RTW among people on sick leave due to MSK disorders. The objectives and hypotheses are presented in detail the statistical analysis plan in Appendix 1. This multi-arm RCT is not designed to compare the two interventions head-to-head, as this would have required an unrealistically large sample size. In addition to analyses of effectiveness of the two interventions, we will conduct health economic analyses, mediation analyses, process evaluation, and exploratory analyses of potential predictors for sick leave due to MSK disorders.

## Methods

### Participants, interventions and outcomes

#### Study setting

This multi-arm RCT (Clinicaltrials identifier: NCT03871712) is work package three (WP3) of the MI-NAV study (https://www.muskhealth.com/minav) consisting of three WPs.

WP1 includes a systematic mapping review [8] and a survey and focus group interview of NAV caseworkers investigating usual NAV case management for individuals on sick leave due to MSK disorders, and experiences of the RTW process. In WP2, we will explore the most accurate screening tool to identify people at a high risk of prolonged sickness absence due to MSK disorder, and investigate MSK health, health-related quality-of-life, health care consumption, and costs across different risk profiles in individuals on sick leave due to MSK disorders [19]. The first phase of WP3 will be an *internal* pilot trial including recruitment of the first 100 study participants. We will assess whether the recruitment protocol, and the randomization procedure, and that the interventions and follow-ups will run as planned. If we do not need to do changes in these parts after the pilot study, data from the first 100 recruited will be included in the main trial. If major changes to any of the recruitment, randomization or intervention procedures will be needed, we will do the protocol changes before starting the RCT or stop the trial if it turns out to not be feasible.

The RCT is a collaboration project between the MUSK Health research group at Oslo Metropolitan University and NAV and will be conducted in the South-Eastern region of Norway. The recruitment of participants will be conducted at the NAV Directorate in Oslo, Norway. The RCT will be reported according to the CONSORT extension statement for multi-arm trials [20] and this protocol is reported according to the Standard Protocol Items: Recommendations for Interventional Trials (SPIRIT) [21].

#### Eligibility criteria

People on sick leave because of any MSK disorder will be eligible for inclusion if they are 18–67 years old and have been on sick leave for > 7 weeks. Individuals with full or part-time positions will be eligible if they are on sick leave for at least 50% of their normal working hours, and if they have a job to return to. The exclusion criteria are serious somatic or mental health disorders (e.g. cancer, psychiatric disorders), pregnancy; self-employed workers or freelancers, and insufficient Norwegian or English communication skills to complete participant questionnaires.

#### Interventions

All three arms of this RCT will be offered *usual case management* from NAV caseworkers. The usual case management should ideally follow this timeline: within the first 4 weeks of sick leave, an RTW plan is made by the employer and worker; within 7 weeks, a dialogue meeting between the employee, employer and other relevant stakeholders such as general practitioner (GP), is arranged by the employer. Within week 26 of the sick leave period, NAV arranges a second dialogue meeting between the employee, the employer and in some cases the GP who issued the sick leave.

The participants in the *MI arm* will, in addition, be offered two MI conversations provided by trained NAV caseworkers; the first as soon as possible after random allocation, and the second two weeks later. NAV caseworkers will be educated in MI through a total of 7 intensive course days given by a trained psychologist and a psychiatrist (3 × 2 days + 1 day). In order to avoid contextual contamination, we have strived for collaboration with NAV offices where caseworkers have not used MI in usual case management. The MI education includes the MI principles: a) motivation to change is elicited from the client, and is not imposed upon them by others, b) it is the client’s task to articulate and resolve ambivalence for change, c) direct persuasion is not an effective method for resolving ambivalence, d) adopt a quiet counselling style to elicit information from the client, e) the counsellor is directive in helping the client to examine and resolve ambivalence, and f) readiness to change is not a trait of the client, but a fluctuating result of interpersonal interaction. The NAV case workers will receive a written manual with guidelines on how to conduct the MI in the RCT and will be mentored by a psychologist throughout the intervention period.

The participants in the *SVAI arm* will, in addition to usual case management, be offered 1–2 telephone calls for the low/medium risk group or 3–4 telephone calls and/or face-to-face meetings for the high-risk group. The 10-item Keele STarT MSK tool and the 10-item version of the Örebro MSK Pain Screening Questionnaire Short Form (ÖMPSQ-SF) will be used in combination to stratify the participant into one of two risk groups before group allocation: low/medium risk of long-term sick leave or high risk of long-term sick leave [22]. The Keele STarT MSK tool [15] consists of 10 items (scores range from 0 to 12) that together predict persistent disabling pain and stratifies patients into low, medium or high risk subgroups. The Norwegian version of this tool has been translated and cross-culturally adapted to Norwegian among people on sick leave due to MSK disorders included in a sub-study of the MI-NAV project (manuscript to be published). The short 10-item version of the ÖMPSQ-SF [16] is a screening tool developed to assist in the early identification of those at high risk of work disability. It contains important risk factors for long-term sickness absence due to MSK disorders, including items assessing pain catastrophizing and fear-avoidance beliefs, depression, anxiety, and RTW expectancy. To ensure both high sensitivity and specificity, we will use both these instruments to screen the participants to low/medium or high risk of long-term sick leave groups. In the current RCT, participants will be allocated to the high-risk subgroup if they have ≥9 points (out of 12) on the Keele STarT MSK tool and ≥ 60 points (out of 100) on the ÖMPSQ-SF at baseline. These cut offs are based on preliminary data from WP2 of this project [19].

Eight physiotherapists will be trained over a five-day course to provide *SVAI*. They will give the intervention to the participants in the SVAI arm as follows; the first contact should ideally be within a week after allocation. This intervention will stop by week 26 of the sick leave period or if the participant has returned to work for more than 4 weeks in the same working hours as they had before going on sick leave. The physiotherapists will receive monthly mentoring during the intervention period by the course instructor (GS) and two of the researchers (FA and AT). The SVAI is based on the SWAP trial from the UK [23], but delivered as a stratified intervention (see below) and adapted to fit a Norwegian context. The intervention emphasizes the assessment and problem solving of modifiable health and work-related obstacles to RTW, in collaboration with key stakeholders. The physiotherapists follow a structured protocol during the intervention sessions, in which they also document aspects of the intervention and obstacles to RTW. The SVAI will be described in greater detail in a separate process evaluation paper, but a brief description is given here. In this arm of the trial participants will be offered treatment that is matched to their risk subgroup as follows:

- Participants at low/medium risk of long-term sick leave (according to the screening) will be offered one to two phone calls (lasting up to 1 h) containing evidence-based advice on best current care for the management of MSK pain and information and support to overcome obstacles associated with RTW. If the participant requires further support to address modifiable obstacles to RTW (e.g. if he/she is not confident to RTW and/or can not specify when they will return), a second phone consultation can be conducted.
- Participants at high-risk of long-term sick leave will be offered three to four sessions comprising evidence-based advice on the management of MSK pain and information and support to overcome obstacles associated with RTW. The first session will be conducted over the telephone, but the following sessions can either be by phone or face-to-face, including an optional worksite visit. If the participant consents, written evidence-based information can be sent to key stakeholders (the worker, employer, GP and other healthcare professionals) in order to inform all involved about the intervention and encourage the coordination of their efforts to help the participant overcome obstacles to RTW.

#### Outcomes

Baseline demographic data includes sociodemographic and background information about sex, age, level of education, marital status, first language, smoking, current workability, use of painkillers and non-steroid anti-inflammatory drugs (NSAIDs), sleeping medicine, and physical activity level last week. Health literacy will be assessed at baseline using the Health literacy Scale Norwegian Questionnaire 12 (HLS-N-Q12) [24], a 12 item questionnaire covering: finding information about the diagnosis, assessing pros and cons with different treatments, following health instructions, assessing validity of information, finding and understanding information, and taking decisions for improved health. Sickness absence data will be delivered by NAV from registries. We will apply for recorded data on the use of health care in the National Patient Registry (NPR) and the national registries for secondary and primary health care in Norway.

#### Primary outcome

The primary outcome is the number of sickness absence days from baseline assessment date until the 6-month follow-up (main endpoint). We will convert time on sick leave to days of sickness absence by combining information from different national registries including information on sick leave payments, sick leave certificates, work assessment allowance (AAP), disability pensions and employment percentage. We will convert time on graded sick leave to actual days away from work (according to a five-day work week) adjusted for employment percentage. Any increase in disability pension during follow-up will be counted as sick leave. AAP is paid every 14 days and adjusted for how much a person has worked in this period. To compute days of sickness absence for people receiving AAP we will divide the actual payment by the maximum possible payment, to estimate the proportion of AAP that a given person has received. This will then be transformed into days of sickness absence.

#### Secondary outcomes

1. The number of sickness absence days from baseline until the 12-month follow-up (registry data).
2. Time until full sustained RTW (first 4-week period of 50–100% return to original employment percentage without relapse) during 12 months of follow-up (registry data).
3. The proportions of participants receiving sick leave benefits during the 12 months of follow-up (registry data).
4. Quality-adjusted life years (QALYs) measured by the EuroQol-5 Dimentions-5 Levels (EQ-5D-5L).
5. MSK health at the 12-month follow-up will be assessed by the MSK Health Questionnaire (MSK-HQ). MSK-HQ has 15 items and is a recently developed questionnaire that captures key outcomes that have shown to be highly relevant to patients across a broad variety of MSK disorders presentations. It has undergone initial psychometric testing in four different MSK cohorts and demonstrated high completion rates, excellent test-retest reliability and strong convergent validity with other disease-specific outcomes [22].

#### Sample size

The sample size was calculated based on the estimates from two previous trials, the first by Linton and colleagues [14] which was conducted in Sweden in a welfare system similar to the Norwegian one, and the second, the SWAP trial by Wynne-Jones and colleagues [17] in the UK. Linton et al. found a difference of 11.3 sickness absence days in favor of the intervention group (MI and problem-solving skills for the patient and their work supervisor) compared to treatment as usual at 6 months, and the SWAP trial found a difference between intervention and usual primary care of 5.1 days after 4 months. As there was no information about the standard deviation (SD) in the study by Linton et al., we estimated it using their results. Thus, we calculated a SD of 27.4 for the difference of 11.3 days. The total number of sick days was calculated from inclusion of the study until 6 months later. Considering a difference of 10 days (SD 28) to be clinically relevant between the usual care management alone versus usual care management plus MI and between usual case management alone versus usual case management plus SVAI arm, and with statistical power of 80% and significance level of 5%, we would need 125 individuals per arm, in total 375 for this multi-arm RCT. Considering adjustment for expected skewed data for the primary outcome (days of sick leave) with a factor of 1.1, we estimated a sample size of 413 participants. Since the primary work-related outcomes are based on registry data, we anticipate there will be very little missing data, hence, we adjusted for only 5% loss to follow-up, and so the estimate is 434 participants, and we decided to include 450 participants in total (150 in each trial arm). To reduce loss to follow-up for secondary outcomes, we will use several initiatives such as brief questionnaires, sending reminders, and provide English questionnaires for those who do not speak/write Norwegian.

#### Identification, recruitment and randomization

Participants will be identified by the NAV Directorate by weekly updated lists of people on sick leave due to MSK disorders for > 7 weeks in the South-Eastern region of Norway. Eligible participants will then be contacted by telephone and asked if they would like to participate and then screened for inclusion and exclusion criteria. If the eligible individual confirms participation via telephone, an electronic link with informed consent and questionnaires will be completed. We will administer the questionnaires to all participants electronically before allocation to groups (baseline), and at 3-, 6-, 9-, and 12-month thereafter. The timeline is illustrated in Table 1.

**Table 1:** The schedule of enrolment, interventions, and assessments

	STUDY PERIOD						
	Enrolment	Allocation	Post-allocation (months)				Long-term
**TIMEPOINT**	*-t*_*1*_	0	1	3	*6*	*9*	*1 year*
**ENROLMENT**							
Eligibility screen	X						
Informed consent	X						
Allocation		X					
**INTERVENTIONS**							
Motivational interviewing			X				
Stratified vocational advice			X	(X)			
Usual case management	X		(X)	(X)	(X)	(X)	
QUESTIONNAIRES							
Education, marital status, smoking, work expectancy, HLS-N-Q12	X						
Work ability, conflicts and well-being at work, medications, physical activity level, STarT MSK, Örebro Musculoskeletal Pain Screening Questionnaire Short Form, Coping from (ÖMPSQ full version), MSK-HQ, iPCQ, work situation, EQ-5D-5L	X			X	X	X	X
Global rating of change, medical/health treatment last 3 months				X	X	X	X
Return to work days							X

*HLS-N-Q12* Health Literacy Scale Questionnaire, *STarT MSK* Start Musculoskeletal tool, *MSK-HQ* Musculoskeletal Health Questionnaire, *iPCQ* the Institute for Medical Technology Assessment Productivity Cost Questionnaire, *EQ-5D-5L* Euro Quality of Life 5 Dimensions 5 Levels

**Fig. 1:**
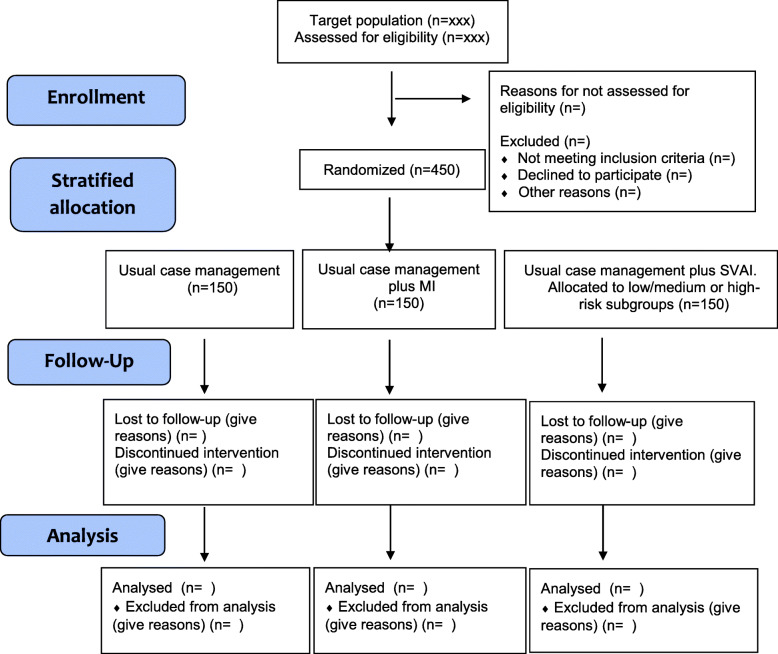
Flow chart of study participants in the randomized controlled trial

Two computer-generated randomization lists will be prepared by a blinded statistician, who will not be involved in the allocation sequence: One list is prepared for the high-risk subgroup and one list for the low/medium risk subgroup. After stratification for high and low/medium risk, a random number will be generated from a range of 1 to 3 using random number generator implemented in Matlab software (a 1:1:1 allocation within each strata). The lists are unavailable for everyone, except for the person who allocate the participants (TR). The person who will allocate the participants will contact the NAV case workers and the physiotherapists offering the interventions by phone and will not be a part of the recruitment ensuring concealed allocation. A flow chart is depicted in Fig. 1.

#### Blinding

The researchers (assessors and statistician) will be blinded to the allocation of participants, and the analyses will be blinded. Participants, NAV caseworkers and physiotherapists delivering the interventions will not be blinded.

#### Statistical analyses

A statistical analysis plan (SAP) for the primary and secondary outcome analyses, and the economic evaluation, is presented in Appendix I. A separate SAP for the mediation analyses and for the exploratory predictor analyses will be reported. The analysis will follow the intention to treat principle and are described in detail in the SAP. Cost-utility, cost-effectiveness and cost-benefit analyses will be performed from a health and societal perspective. The time horizon is 12 months. To measure utilities the EQ-5D-5L utility index will be used [25]. The EQ-5D-5L is a generic and preference-weighted measure of health-related quality-of-life based on five dimensions: mobility, self-care, activities of daily life, pain, and anxiety and/or depression. For each dimension, the patient assesses five possible levels of problems (from no to severe) [25]. The participants complete the EQ-5D-5L at baseline, and at the 3-, 6-, 9- and 12-months follow-ups. Health gains will be expressed as QALYs. Healthcare utilization and work loss will be based upon public registry data. In addition to public registry data on sickness absence, production loss will also be measured by the Institute for Medical Technology Assessment Productivity Cost Questionnaire (iPCQ) [26], which assesses absenteeism, presenteeism and unpaid work. The iPCQ has been translated and culturally adapted to Norwegian and found to have good measurement properties when used among Norwegian patients with long-term MSK disorders [27].

To compare participants with eligible people from the general Norwegian population and thus to evaluate representativeness of our sample, we will use anonymized data from NAV on sociodemographic and work-related variables (sex, age, diagnosis, graded disability pensions, type of occupation and employment percentage. These analyses will be conducted across different sub-populations; 1) between trial participants and eligible people from the same geographic area in Norway; 2) between trial participants and eligible people from the whole of Norway; 3) between trial participants from the present MI-NAV project and trial participants in a similar ongoing trial in Trondheim [28].

#### Process evaluations of MI and SVAI

We will conduct process evaluations of both the MI and SVAI interventions using a mixed-methods design. The usual case management will be described in WP1 of the MI-NAV Study. The process evaluation involves investigating which factors promote and restrain the implementation, and acceptability and maintenance of the two interventions. In addition to the fidelity and adherence scorings described below, the content of audio-recordings and recorded data from the MI- and SVAI-conversations will be explored by qualitative methods. Whether the interventions are delivered as intended will be assessed through audio-recordings of approximately 10% of the intervention sessions (in the two intervention arms). Recordings of the conversations between the participants and either the NAV caseworkers or physiotherapists will be done throughout the intervention period. In addition, we will log the number of participants per NAV caseworker and SVAI physiotherapist so that those who have the most participants will perform the most recordings. The fidelity of the MI conversations will be scored and coded using the Motivational Interviewing Treatment Integrity Code version 4 (MITI 4) [29]. This scoring will be done by trained people at the KoRus MI-analysis Center in Bergen (https://www.korusbergen.no/motiverende-samtale/mi-analyse/), who are not part of the research group. We will document and report mean and percentages of MITI scores, representing degree of MI competence, as well as more detailed analyses of the change in MITI scores over time.

We will record adherence in the two intervention arms as numbers and percentages of participants who have received the intervention according to the protocols. For the MI arm, adherence will be defined as receiving two sessions in which at least one of them should be face-to-face meeting. For the SVAI intervention adherence will be defined as receiving one or two sessions (phone calls only) in the low/medium-risk group and three or four sessions either by phone or face-to-face in the high-risk group. In addition, a SVAI log should be completed, indicating that the structured intervention protocol has been followed. The SVAI log is a document kept by the SVAI physiotherapists for each participant in that arm containing themes that the physiotherapist should ask about during the conversation (i.e. obstacles for RTW, RTW plan, contact with employer, health situation, work situation). We will also report the number of face-to-face sessions and the number of workplace meetings in the high-risk subgroup.

#### Mediators

The two possible mediators of the association between the interventions and number of sickness absence days at 6 months, workability and RTW expectancy, will be assessed as follows: Workability will be assessed by one single item from the Finnish Work Ability Index (WAI) [30] recording “current workability compared with the lifetime best” on a 0–10 numerical rating scale. Work expectancy assessed by one question from the ÖMPSQ-SF (“In your estimation, what are the chances you will be working your normal duties in 3 months”) and a question on how long they think they will be on sick leave (< 2 months; 2–4 months; 4–10 months, or > 10 months).

#### Predictors

To evaluate whether any subgroup of participants have a different treatment effect on the primary outcome at 12 months follow-up we will conduct exploratory analyses on predefined predictors (e.g. the risk groups). A separate SAP will be worked out for these analyses and published at clinicaltrials.gov.

#### User involvement

In the planning of the RCT, we consulted an established user panel^1^ with representatives from different patient organizations for people with MSK disorders. They gave feedback related to the relevance, aim and conduction of the study. The user representatives considered the study to be important and of high relevance to people with MSK disorders. Members from the user panel helped with the wording of the information letter and the development of the recruitment telephone script. We plan to involve users in interpreting the data, drafting the manuscripts and disseminating the results.

#### Ethical considerations

The study protocol was submitted to the Regional Committees for Medical and Health Research Ethics for health research. They concluded that the study did not need approval by the Committee since the study does not generate new health research (2018/1326/REK sør-øst A). The Norwegian Center for Research Data has assessed and approved the project. The study will be conducted according to the Helsinki declaration. Data will be stored on Services for Sensitive Data (TSD) at the University of Oslo on secured research servers with access only for researchers directly involved in the project.

## Discussion

The Norwegian Directorate of Health encourages health care providers to use MI to support health behavior change, and MI is used in NAV to facilitate RTW despite little research supporting the use of MI in people with MSK disorders [8]. To be able to give evidence-based recommendations on the effectiveness and cost-effectiveness of MI on RTW, there is a need for large RCTs. Whilst other vocational advice interventions have shown promising results in improving RTW outcomes in individuals with MSK disorders [17, 31], a stratified vocational advice intervention provided by physiotherapists, as included in this RCT, has not been studied previously. In this multi-arm RCT all participants receive usual case management by NAV and the two intervention groups receive additionally either MI or SVAI. This design enables us to examine the additional effect of the vocational interventions to the usual case management, however, we have not designed the study to compare the two interventions head-to-head, as this would have required an unrealistically large sample size. The primary outcome is the number of sick leave days from inclusion to the 6-month follow-up, and the sample size calculation is based on this follow-up time-point with an expected difference between the intervention groups and the usual case management group of 10 days.

Experienced professionals will educate both the NAV caseworkers and the SVAI physiotherapists to provide the interventions. However, it is difficult to conduct MI correctly with little experience [6]. Therefore, we will pay close attention to how the NAV caseworkers and the SVAI physiotherapists will use and develop their skills in the interventions through audiotaping the sessions, score the MI interventions, and conduct monthly mentoring and supervision sessions.

Conducting RCTs with people on sick leave is dependent upon the willingness of these people to participate in research. A previous RCT on the effectiveness of inpatient multicomponent occupational rehabilitation found that less than 10% of eligible participants accepted the trial invitation [32]. Thus, we have tried to keep the questionnaires as short as possible to prevent missing data. We will have data from all the participants on the main outcome from NAV registries and we will perform the analyses according to the intention-to-treat principle to reduce the impact of attrition bias. Nevertheless, we need more knowledge of the group that is willing to participate in research as compared to those who do not participate. This will be addressed by comparing registry data of those who are willing and those who do not participate in this RCT and the other similar ongoing RCT in Norway [28]. The results of the RCT will provide evidence of the clinical and cost-effectiveness of MI and SVAI in addition to usual NAV case management in Norway.

## Supplementary information

**Additional file 1:Appendix I:** Statistical analysis plan for the MI-NAV study: Study protocol for a randomized controlled trial of the effectiveness of adding motivational interviewing or stratified vocational advice intervention to usual case management on return to work for people with musculoskeletal disorders. The MI-NAV study.

## Data Availability

Not applicable for this publication.

## Abbreviations

AAP: Work Assessment allowance
EQ-5D-5L: Euro Quality of life 5 dimentions 5 levels
GP: General practitioner
HLS-N-Q12: Health literacy Scale Norwegian Questionnaire 12
iPCQ: Institute for Medical Technology Assessment Productivity Cost Questionnaire
MI: Motivational interviewing
MITI: Motivational Interviewing Treatment Integrity Code
MSK: Musculoskeletal disorders
MSK-HQ: MSK Health Questionnaire
NAV: Norwegian Labour and Welfare Administration
NPR: National Patient Registry
QALY: Quality-adjusted life years
RCT: Randomized controlled trial
RTW: Return to work
SAP: Statistical analysis plan
SD: Standard deviation
SPIRIT: Standard Protocol Items: Recommendations for Interventional Trials
SVAI: Stratified Vocational Advice Intervention
SWAP: Study of Work and Pain
TSD: Services for sensitive data
UK: United Kingdom
WP: Work package
ÖMPSQ-SF: Örebro MSK Pain Screening Questionnaire Short Form

## Footnotes

1 https://oslo-universitetssykehus.no/avdelinger/nevroklinikken/forskning-og-utvikling-nevroklinikken/forsknings-og-formidlingsenheten-for-muskelskjeletthelse-formi/formi-brukermedvirkerbase
