# Supplementary material for: Study protocol for a randomized controlled trial of the effectiveness of adding motivational interviewing or stratified vocational advice intervention to usual case management on return to work for people with musculoskeletal disorders. The MI-NAV study
Source: BMC Musculoskelet Disord. 2020 Jul 28;21:496. doi: 10.1186/s12891-020-03475-z (PMC7385885; doi:10.1186/s12891-020-03475-z)
Supplement: Supplementary file 1 — Additional file 1:Appendix I: Statistical analysis plan for the MI-NAV study: Study protocol for a randomized controlled trial of the effectiveness of adding motivational interviewing or stratified vocational advice intervention to usual case management on return to work for people with musculoskeletal disorders. The MI-NAV study. [file 12891_2020_3475_MOESM1_ESM.pdf]

APPENDIX I: STATISTICAL ANALYSIS PLAN FOR THE MI-NAV STUDY: A RANDOMIZED CONTROLLED TRIAL OF THE EFFECTIVENESS OF ADDING MOTIVATIONAL INTERVIEWING OR STRATIFIED VOCATIONAL ADVICE INTERVENTION TO USUAL CASE MANAGEMENT ON RETURN TO WORK FOR PEOPLE WITH MUSCULOSKELETAL DISORDERS

**Clinical trials registration number:**	NCT03871712
**SAP version:**	1th version
**Date:**	27.05.2020
**Protocol version:**	1th version

This document is a statistical analysis plan (SAP) for the MI-NAV study and is a supplement to the MI-NAV study protocol. We have followed the guidelines for the content of statistical analysis plans in clinical trials (1), all the recommended items are described either in this SAP or in the MI-NAV study protocol.

Administrative executive board

Hege Bentzen
Margreth Grotle
Therese Sundell

Contributors to SAP

Fiona Aanesen,[1] Britt Elin Øiestad,[1] Ida Løchting,[1,2] Kjersti Storheim,[1,2] Alexander Tingulstad,[1] Tarjei Rysstad,[1] Milada C Småstuen,[1,3] Anne Therese Tveter,[1,4] Gail Sowden,[5,6] Gwenllian Wynne-Jones,[5,7] Egil A Fors,[8,9] Maurits van Tulder,[10] Rigmor C Berg,[11,12] Nadine E Foster,[5] Margreth Grotle,[1,2]

[1] Department of Physiotherapy, Oslo Metropolitan University, Norway
[2] Research and Communication Unit for Musculoskeletal Health (FORMI), Oslo University Hospital, Norway
[3] Department of nursing and health promotion, Oslo Metropolitan University, Norway
[4] National Advisor Unit on Rehabilitation in Rheumatology, Department of Rheumatology, Diakonhjemmet Hospital, Oslo, Norway
[5] School of Primary, Community & Social Care, Keele University, Keele, UK
[6] Connect Health, Newcastle upon Tyne, UK
[7] School of Nursing and Midwifery, Faculty of Medicine and Health Sciences, Keele University, Keele, UK
[8] General Practice Research Unit, Department of Public Health and Nursing Faculty of Medicine and Health Sciences, Norwegian University of Science and Technology (NTNU), Trondheim, Norway
[10] Department Health Sciences, Faculty of Science, Vrije Universiteit Amsterdam, the Netherlands
[11] Norwegian Institute of Public Health, Oslo, Norway
[12] Department of Community Medicine, The University of Tromsø, Tromsø, Norway

Signatures

I hereby declare that I have reviewed and approved the statistical analysis plan for the MI-NAV study:

Name	Role	Date, signature
Fiona Aanesen	Main author of SAP	27.05.2020 Fiona Aanesen
Britt Elin Øiestad	Primary investigator	28.05.2020 Britt Elin Øiestad
Margreth Grotle	Coordinating investigator	27.05.2020 Margreth Grotle
Milada C Småstuen	Senior statistician responsible for statistical methodology	Milada C. Småstuen

List of abbreviations

Abbreviation	Explanation
AAP	Work assessment allowance
BMI	Body mass index
CI	Confidence interval
EQ-5D-5L	The EuroQol 5 dimension, 5 level questionnaire
HLS-Q12	European health literacy survey questionnaire, short version
MI	Motivational interviewing
MSK	Musculoskeletal
MSK-HQ	Musculoskeletal health questionnaire
NAV	Norwegian labour and welfare administration
ÖMPSQ-SF	Örebro Musculoskeletal Pain Screening Questionnaire Short Form
RTW	Return to work
SAP	Statistical analysis plan
SD	Standard deviation
STarT MSK	The Keele STarT MSK Tool
SVAI	Stratified vocational advice intervention
WAI	Work ability index

STUDY OBJECTIVES

The main objective of the MI-NAV study is to compare the effectiveness and cost-effectiveness of usual case management alone with usual case management plus motivational interviewing (MI) or usual case management plus stratified vocational advice intervention (SVAI), on return to work (RTW) among people on sick leave due to musculoskeletal (MSK) disorders. All groups will receive usual case management provided by the Norwegian Labour and Welfare Administration (NAV). The study participants in the two intervention arms will in addition receive either MI delivered by NAV caseworkers or SVAI delivered by physiotherapists. The effectiveness of each intervention (MI or SVAI) will be compared to usual case management from NAV (control group). The study rationale, methodology, assessments of outcomes, adherence and fidelity are described in detail in the MI-NAV study protocol.

RESEARCH QUESTIONS AND HYPOTHESES

Research questions (RQ)	Null hypotheses (H)
**Primary**	
RQ 1a<br>Is there a difference between usual case management plus *MI* and usual case management alone in reducing sickness absence days at 6 months follow-up among individuals who have been on sick leave for >7 weeks due to a musculoskeletal disorder?	H 1a<br>There is no difference in number of sickness absence days between participants who receive usual case management plus *MI* compared to those who receive usual case management alone at 6 months follow-up.
RQ 1b<br>Is there a difference between usual case management plus *SVAI* and usual case management alone in reducing sickness absence days at 6 months follow-up among individuals who have been on sick leave for >7 weeks due to a musculoskeletal disorder?	H 1b<br>There is no difference in number of sickness absence days between participants who receive usual case management plus *SVAI* compared to those who receive usual case management alone at 6 months follow-up.
**Secondary**	
RQ 2a<br>Is there a difference between usual case management plus *MI* and usual case management alone in reducing sickness absence days at 12 months follow-up among individuals who have been on sick leave for >7 weeks due to a musculoskeletal disorder?	H 2a<br>There is no difference in number of sickness absence days between participants who receive usual case management plus *MI* compared to those who receive usual case management alone at 12 months follow-up.
RQ 2b<br>Is there a difference between usual case management plus *SVAI* and usual case management alone in reducing sickness absence days at 12 months follow-up among individuals who have been on sick leave for >7 weeks due to a musculoskeletal disorder?	H 2b<br>There is no difference in number of sickness absence days between participants who receive usual case management plus *SVAI* compared to those who receive usual case management alone at 12 months follow-up.
RQ 3a<br>Is there a difference between usual case management plus *MI* and usual case management alone in time until sustained RTW during 12 months follow-up among individuals who have been on sick leave for >7 weeks due to a musculoskeletal disorder?	H 3a<br>There is no difference in time until first sustained RTW between participants who receive usual case management plus *MI* compared to those who receive usual case management alone during 12 months follow-up.
RQ 3b<br>Is there a difference between usual case management plus *SVAI* and usual case management alone in time until sustained RTW during 12 months of follow-up among individuals who have been on sick leave for >7 weeks due to a musculoskeletal disorder?	H 3b<br>There is no difference in time until first sustained RTW between participants who receive usual case management plus *SVAI* compared to those who receive usual case management alone during 12 months of follow-up.
RQ 4a<br>Is there a difference in the proportions of participants who receive sick leave benefits each month during 12 months of follow-up between usual case management plus *MI* compared to usual case management alone?	H 4a<br>There is no difference in the proportions of participants who receive sick leave benefits each month between usual case management plus *MI* compared to usual case management alone during 12 months of follow-up.

RQ 4b<br>Is there a difference in the proportion of individuals who receive sick leave benefits each month during 12 months of follow-up between usual case management plus *SVAI* compared to usual case management alone?	H 4b<br>There is no difference in the proportions of individuals receiving sick leave benefits each month between usual case management plus *SVAI* compared to usual case management alone during 12 months of follow-up.
RQ 5a<br>Is there a difference in cost-effectiveness, cost-utility and cost-benefit during 6 months of follow-up between individuals on sick leave with musculoskeletal disorders who receive usual case management plus *MI* compared to those who receive usual case management alone?<br><br>RQ 5b<br>Is there a difference in cost-effectiveness, cost-utility and cost-benefit during 6 months of follow-up between individuals on sick leave with musculoskeletal disorders who receive usual case management plus *SVAI* compared to those who receive usual case management alone?<br><br>RQ 6a<br>Is there a difference in cost-effectiveness, cost-utility and cost-benefit during 12 months of follow-up between individuals on sick leave with musculoskeletal disorders who receive usual case management plus *MI* compared to those who receive usual case management alone?<br><br>RQ 6b<br>Is there a difference in cost-effectiveness, cost-utility and cost-benefit during 12 months of follow-up between individuals on sick leave with musculoskeletal disorders who receive usual case management plus *SVAI* compared to those who receive usual case management alone?	H 5a<br>There is no difference in cost-effectiveness, cost-utility and cost-benefit between usual case management plus *MI* compared to usual case management alone during 6 months of follow up.<br><br>H 5b<br>There is no difference in cost-effectiveness, cost-utility and cost-benefit between usual case management plus *SVAI* compared to usual case management alone during 6 months of follow up.<br><br>H 6a<br>There is no difference in cost-effectiveness, cost-utility and cost-benefit between usual case management plus *MI* compared to usual case management alone during 12 months of follow up.<br><br>H 6b<br>There is no difference in cost-effectiveness, cost-utility and cost-benefit between usual case management plus *SVAI* compared to usual case management alone during 12 months of follow up.
RQ 7a<br>Is there a difference in musculoskeletal health during 12 months of follow up between individuals on sick leave with musculoskeletal disorders who receive usual case management plus MI compared to those who receive usual case management alone?<br><br>RQ 7b<br>Is there a difference in musculoskeletal health during 12 months of follow up between individuals on sick leave with musculoskeletal disorders who receive usual case management plus SVAI compared to those who receive usual case management alone?	H 7a<br>There is no difference in musculoskeletal health between participants who receive usual case management plus *MI* compared to those who receive usual case management alone during 12 months of follow-up.<br><br>H 7b<br>There is no difference in musculoskeletal health between participants who receive usual case management plus *SVAI* compared to those who receive usual case management alone during 12 months of follow-up.

MI: motivational interviewing, SVAI: stratified vocational advice intervention, NAV: Norwegian Labour and Welfare Administration, RTW: return to work.

STUDY METHODS

The trial is a multi-arm randomized controlled trial containing an *internal* pilot study consisting of the first 100 study participants. The aim of the internal pilot is to test the procedures for recruitment, randomisation and intervention delivery. No pre-specified progression criteria are set but the recruitment rate will be monitored during the pilot period. If major changes are made to the study protocol the RCT will start after the changes are made, if no major changes are needed the data from the pilot study will be included in the main trial.

The allocation of participants to the three groups: usual case management, usual case management + MI or usual case management + SVAI, is determined by block randomization stratified by risk group for long-term sick leave. A detailed description of the screening tools including cut-off values and measurement tools for other variables is presented in the MI-NAV study protocol. This protocol describes the randomization method, sample size calculations, recruitment strategy and eligibility criteria.

Inclusion and dropout

Information about recruitment, inclusion, exclusion, dropouts and loss to follow-up will be reported in a flow diagram in the main article as illustrated in Figure 1 in the study protocol.

Outcomes

Primary outcome (I)

The number of sickness absence days from baseline assessment date until the 6-month follow-up (data retrieved from national registers).

Secondary outcomes (II)

a. The number of sickness absence days from baseline until the 12-month follow-up (data from national registers).
b. Time until full sustained RTW during 12 months of follow-up, defined as first 4-week period of 50-100% return to original employment percentage without relapse (data from national registers).
c. The proportions of participants receiving sick leave benefits during the 12 months of follow-up (measured as repeated events assessed with data from national registers).
d. Quality-adjusted life years (QUALYs) measured by the EuroQol-5 Dimensions-5 Levels (EQ-5D-5L).
e. Musculoskeletal health at the 12 months follow-up (assessed with the Musculoskeletal Health Questionnaire (MSK-HQ) at baseline, 3, 6, 9 and 12 months follow up).

Analyses

All analyses will be performed by a Ph.D.-student in collaboration with a statistician and the co-authors using the statistical software SPSS version 25 and STATA version 15. The outcomes will be analysed separately for the ‘usual case management + MI arm’ compared to the ‘usual case management alone arm’, and the ‘usual case management + SVAI arm’

compared to the 'usual case management alone arm'. All relevant statistical tests will be two-sided. A p-value of <0.05 will be considered statistically significant and all point estimates will be reported with 95% confidence intervals (CI).

The analyses will be performed at the 6 and 12 months follow-up. No interim analyses are planned, and the trial will continue until we have included 450 participants in the study. The primary and secondary analyses will be performed according to the intention-to-treat principle. The intention-to-treat principle is defined as follows: all included participants randomized to a given arm will be analysed, thus including participants who did not receive the interventions.

Preliminary analyses

After checking the data for errors and cleaning the data file we will perform descriptive statistics. Categorical variables will be described with frequency tables including percentages. We will describe continuous variables with medians and range (for data with skewed distribution) and means and standard deviations (SD) if the data is normally distributed. If there are many missing values, we will perform Littles's missing completely at random test to assess if the missing values are random or not. We will check if the continuous variables are normally distributed by inspecting the shape of the distributions using histograms, we will also check the skewness and kurtosis values and perform a Kolmogorov-Smirnov test for normality. We will check for outliers by inspecting histograms and boxplots, the effect of any outliers will be assessed by comparing a 5% trimmed mean to the total mean.

Baseline characteristics

Baseline characteristics will be presented for participants in the three trial arms, the variables which will be described are presented in Table 1. Continuous variables will be reported as means with SD for normally distributed data, and with medians and ranges for variables with skewed distributions. Categorical variables will be reported as counts and percentages.

Covariates in multiple analyses of outcomes I, II a, b and c

The following factors are considered as possible confounding variables associated with RTW for people on sick leave with musculoskeletal disorders: age, sex, education level, sick leave previous year, workability, musculoskeletal health, risk of work disability, physical activity and employer follow-up. If any of these variables are not equally distributed in the 'usual case management + MI' arm compared to 'usual cases management alone' arm, or 'usual case management + SVAI' arm compared to 'usual case management alone' arm at baseline, we will include them as covariates in the multiple analyses of the RTW outcomes to control for confounding.

I. Primary analyses

The analyses of the primary outcome will compare the effectiveness of each of the two interventions (the addition of either MI or SVAI) versus usual case management at 6 months follow-up. The number of sickness absence days is not likely to be normally distributed and will be evaluated with the Mann–Whitney *U* test, if the number of sickness absence days is normally distributed we will use t-tests. We will report the median value and range or the

mean and standard deviation and the p-value from the Mann-Whitney U or t-tests, and between group differences. All analyses will be reported for ‘usual case management + MI’ compared to ‘usual case management alone’ and ‘usual case management + SVAI’ compared to ‘usual case management alone’.

If the groups are not balanced on all possible confounders, we will fit linear mixed models with possible confounders entered as fixed effects and report the unstandardized regression coefficients (B-values) with standard errors and 95% confidence intervals. We will first check if the assumptions for linear regressions are met, including linear relationship between the independent and dependent variable, normally distributed residuals, no multicollinearity between the independent variables, homoscedasticity and independent residuals. These assumptions will be checked by inspecting the normal probability plot and residual scatterplots to check the residuals for normality, linearity and homoscedasticity and variance inflation factor values to check for multicollinearity between the independent variables. If the assumptions for linear regressions are not met, we will perform ordinal logistic regressions. We will group sickness absence days into tertiles based on the distribution in our data and use these three groups as the outcome measure in the analyses.

<u>II. Secondary analyses</u>

a. The analyses for outcome II a) will be identical to the analyses for the main outcome but with data on number of sickness absence days at 12 months follow-up.

b. Time until sustainable return to work will be evaluated with Kaplan Meier survival analysis and the groups will be compared using the log rank test. Time will be calculated as number of months from baseline to participants’ “full sustainable return to work” (as defined in outcome II b) or to the end of 12 months follow-up, whatever comes first. Crude survival curves and p-values from the log rank tests and median time until sustainable return to work with 95% CI will be presented for both intervention arms compared to usual follow-up. We will estimate hazard ratios for return to work using Cox proportional hazard model. The proportionality hazard assumption will be checked using the Schoenfeld residual test. If any of the possible confounding variables are not equally distributed in the control and intervention arms at baseline, we will include these variables in an adjusted Cox regression analysis. We will also include risk group as a covariate to avoid overestimating the variance. Possible differences between the treatment groups will be expressed as hazard ratios with 95% CI.

c. The odds for receiving benefits each month during 12 months follow-up will be analysed with General Mixed Model (GLM) with logit link. We will present the results for the groups over the time frame graphically and present the odds for receiving benefits in each intervention arm compared to usual case management.

d. Cost-effectiveness, cost-utility, and cost-benefit will be analysed from a societal and health perspective. Intervention costs will be calculated based on a micro-costing approach, and will also include training and mentoring costs of the two interventions. Health care utilization and costs will be retrieved from national registers: The Norwegian Health Economics Administration (Helfo) and the Norwegian Patient

Registry. Productivity loss due to sickness absence will be calculated for each follow-up period and adjusted for part-time work (employment rate), as well as percentage of sick leave in the period. The costs of productivity loss will be estimated as the number of days absent from work multiplied by the average wage rate in Norway by sex. Costs for absence from work will be estimated from official statistics of average wage by sex and age groups. Health gains will be expressed as quality-adjusted life years (QALYs), which will be derived from the EQ-5D-5L utility scores. We will use the UK tariff for valuing health-related quality of life, as a Norwegian tariff is not available. QALYs range from -0.59 to 1, where 1 corresponds to perfect health, and -0.59 to worst imaginable health. The willingness-to-pay threshold will be based on the Norwegian governmental report No. 34 to the parliament with a value of NOK 275,000 (Euro (€) 27,500/USD 35,628) per QALY (2).

The outcome measure in the cost-effectiveness analyses will be sickness absence days, and hence productivity costs will not be included in order to avoid double counting. In the cost-utility analyses, which will include productivity costs when using the societal perspective, QALYs will be used as outcome measure, calculated by combining health-related quality-of-life level and duration (3). In both analyses, the incremental cost-effectiveness ratio (ICER) will be calculated for each of the intervention groups, defined by the incremental costs (costs in the intervention group – costs in the usual care group) relative to QALYs gained (QALYs intervention group – QALYs usual care group). Differences between the 'usual case management + MI' group compared to 'usual case management alone' group and 'usual case management + SVAI' group compared to 'usual case management alone' group in QALYs gained will be estimated using the trapezoidal method (the area under the curve combining utility indexes and time). Uncertainty will be analysed using the bootstrap method with 10 000 replicated datasets. To illustrate the statistical uncertainty surrounding the ICERs, the bootstrapped cost and effect pairs will be plotted on a cost-effectiveness plane (CE plane) with the ICERs on the y-axis and the incremental effects on the x-axis. In the cost-benefit analysis the net societal benefit will be calculated by subtracting the difference in direct cost (cost) between each of the intervention groups and the usual case management group, from the difference in indirect costs (benefits) between the groups. A return on investment will be calculated by dividing the benefit of the intervention by the expenditure of the intervention. In the cost-benefit analysis the net societal benefit will be calculated by subtracting the difference in direct cost (cost) between each of the intervention groups and the usual case management group, from the difference in indirect costs (benefits) between the groups.

e. We will use linear mixed models for repeated measures to analyse the effect of the interventions, on musculoskeletal health measured with the MSK-HQ at 3, 6, 9 and 12 months of follow-up. The effect of each of the two interventions will be compared to usual case management from NAV. The fixed effects in the model will be treatment group, intervention deliverer, time of measurement, and baseline score. The effect of the interventions at each time of follow-up will be estimated with the relevant interaction term (group*time). We will incorporate random intercepts in the model to account for the dependence of repeated measures. A second analysis will

be conducted where we include risk group and possible confounding variables associated with musculoskeletal health which are not equally distributed in the control and intervention arms of the study at baseline: sex, age, BMI, smoking, education level and physical activity.

Sensitivity analyses

We will perform two sensitivity analyses for the main outcome of the trial:

1) The first will be an analysis excluding the first 100 participants included in the pilot period of the trial. This is because the fidelity to the intervention might have improved for the intervention deliverers as they gain practice during the pilot study.
2) The second will be an analysis in which we exclude those who return to work for more than 50% of their normal work hours shortly after inclusion. This sensitivity analysis will be performed because participants will not receive the intervention if they have returned to work in the period between answering the baseline questionnaire and intervention start.

In the health economic analyses the following sensitivity analyses will be carried out:

1) Complete case analysis (without adjustment for missing data in the EQ-5D-5L)
2) Uncertainty of the ICER (incremental cost-effectiveness ratio) will be tested by bootstrapping with 5,000 repetitions (probabilistic sensitivity analysis, PSA).
3) In a multiple one-way sensitivity analysis, the relevant costs and QALYs will be varied 20% below and above the estimates provided in the main analyses. The results will be presented in a Tornado diagram showing the number of one-way sensitivity analyses in one graph.
4) If there are outliers in the material, we will conduct sensitivity analyses without these outliers

Missing data

We anticipate few missing values for the primary outcome and the work-related secondary outcomes a), b) and c), as information will be obtained from the Norwegian national social security system registry where all individuals receiving any form of benefits are registered by their social security number.

If there is a lot of missing data from the questionnaires (including the MSK-HQ and EQ-5D-5L) and we assume that the data are missing at random, we will impute missing values with a multiple imputation model. Further, we will perform a sensitivity analysis and compare the results from the analyses with and without imputation and report if there are differences.

REFERENCES

1. Gamble C, Krishan A, Stocken D, Lewis S, Juszczak E, Doré C, et al. Guidelines for the Content of Statistical Analysis Plans in Clinical Trials. JAMA. 2017;318(23):2337-43.

2. Norwegian Ministry of Health and Care Services. Principles for priority setting in health care. Summary of a white paper on priority setting in the Norwegian health care sector. In: Services NMoHaC, editor. Oslo: Norwegian Ministry of Health and Care Services; 2016.
3. Drummond MF, Sculpher MJ, Torrance GW, O'Brien BJ, Stoddart GL. Methods for the economic evaluation of health care programmes. 3rd ed. Oxford: Oxford University Press; 2005.

Table 1. Baseline characteristics

Variable	N	MI (n=)	SVAI (n=)	Control (n=)
**Mean (SD)/ Median (Range)**				
Age				
BMI [a]				
Physical activity [b]				
Sick leave previous year [c]				
Duration sick-leave [d]				
Musculoskeletal health [e]				
Health literacy [f]				
Workability [g]				
Work satisfaction [h]				
Risk of work disability [i]				
**n (%)**				
Women				
Smokers				
Higher education [j]				
Married or living with partner				
Norwegian as first language				
Smokers				
White-collar workers				
Blue-collar workers				
Work: [k]				
3) Full time work				
4) Part time work ≥50%				
5) Part time work <50%				
Graded disability pension [l]				
On full sick-leave [m]				
Diagnostic groups				
In conflict with employer				
Followed-up by employer [n]				

[a] Body Mass index: kg/m^2
[b] Number of days being physically active previous week
[c] Number of days on sick leave one year prior to inclusion in study
[d] Duration in days of current episode of sick leave
[e] Measured with MSK-HQ (0-56)
[f] Measured with HLS-Q12 (12-72)
[g] Measured with single question from WAI (0-10)
[h] Single question (0-10)
[i] Measured with the ÖMPSQ-SF (0-100)
[j] Higher education: college or university
[k] Employment % in work contract
[l] Individuals who work part time and also receive a graded disability pension
[m] Full sick leave from contracted work hours
[n] Employer had meeting and made follow-up plan with employee

Table 2. Cost categories, units, valuation and unit price

Cost categories	Unit	Valuation	Unit price Euros, €	Unit price NOK	Reference (source)
Direct costs of MI (including training/mentoring)	Per patient	Cost			
Direct costs of SVAI (including training/mentoring)	Per patient	Cost			
Direct costs of usual NAV practice (control)	Per patient	Cost			
Non-opioid medication (NSAIDs: ibuprofen, paracetamol, other A-prescription medicines)	Per daily defined dose	Cost			Pharmacy Selling Price (over-the-counter)
Opioid medication (codein)	Per daily defined dose	Cost			Pharmacy Selling Price
General practitioner	Per visit	Cost			NOMA, general practitioner consultation
Medical specialist	Per visit	Cost			NOMA, Specialist health service consultation (fee*2, + 20 min)
Chiropractor	Per visit	Cost			Norsk Kiropraktorforening estimated average
Physiotherapist	Per visit	Cost			The Norwegian Physiotherapy Association, estimated average
Manual therapist	Per visit	Cost			The Norwegian Physiotherapy Association, estimated average
Acupuncture	Per visit	Cost			Average estimate from private pricelists
Other therapists	Per visit	Cost			Average estimate from private pricelists
Surgery	Per surgery	Cost			DRG215B
Hospitalizations (non-surgery)	Per day	Cost			DRG247 (/2) per patient
Rehabilitation stay (outpatient)	Per day	Cost			UniCare price list, adjusted for health region authority supplements
Production loss (225 work days per year)*	Per day	Wage rate adjusted for age and gender			Statistics Norway
Total healthcare costs					
Production loss (225 work days per year)					
**TOTAL COSTS (healthcare + production loss)**					
